# Seasonality Directs Contrasting Food Collection Behavior and Nutrient Regulation Strategies in Ants

**DOI:** 10.1371/journal.pone.0025407

**Published:** 2011-09-26

**Authors:** Steven C. Cook, Micky D. Eubanks, Roger E. Gold, Spencer T. Behmer

**Affiliations:** Department of Entomology, Texas A&M University, College Station, Texas, United States of America; Vanderbilt University, United States of America

## Abstract

Long-lived animals, including social insects, often display seasonal shifts in foraging behavior. Foraging is ultimately a nutrient consumption exercise, but the effect of seasonality *per se* on changes in foraging behavior, particularly as it relates to nutrient regulation, is poorly understood. Here, we show that field-collected fire ant colonies, returned to the laboratory and maintained under identical photoperiod, temperature, and humidity regimes, and presented with experimental foods that had different protein (p) to carbohydrate (c) ratios, practice summer- and fall-specific foraging behaviors with respect to protein-carbohydrate regulation. Summer colonies increased the amount of food collected as the p:c ratio of their food became increasingly imbalanced, but fall colonies collected similar amounts of food regardless of the p:c ratio of their food. Choice experiments revealed that feeding was non-random, and that both fall and summer ants preferred carbohydrate-biased food. However, ants rarely ate all the food they collected, and their cached or discarded food always contained little carbohydrate relative to protein. From a nutrient regulation strategy, ants consumed most of the carbohydrate they collected, but regulated protein consumption to a similar level, regardless of season. We suggest that varied seasonal food collection behaviors and nutrient regulation strategies may be an adaptation that allows long-lived animals to meet current and future nutrient demands when nutrient-rich foods are abundant (e.g. spring and summer), and to conserve energy and be metabolically more efficient when nutritionally balanced foods are less abundant.

## Introduction

Reproduction, hibernation (diapause), and migration are perhaps the best-known examples of life history events in long-lived animals that are entrained to circannual shifts in photoperiod and related environmental factors (i.e., seasonality *per se*) [1]. As animals shift in and out of these circannually driven life history events [2],[3],[4] they experience correlated shifts in their physiology and behavior [5], [6]. What is less well appreciated and understood is the extent to which seasonality *per se* modifies animal physiology and behavior, particularly foraging behavior.

The two most likely observed seasonal modifications associated with foraging behavior are changes in the amount of food collected, and changes in food preferences. In terms of modifying amounts collected, animals such as squirrels [7] and pika [8] are good examples. They collect summer foods in excess of amounts required for immediate use, and cache this excess for use during winter when food is scarce. In terms of switching food preferences, optimal foraging theory predicts that an animal's foraging decisions should maximize energetic gain [9]. Here animals might switch their food preferences to reflect shifts in the availability of particular foods (e.g., [10]). Alternatively, preference switches might indicate active regulation of nutrient intake, despite the relative abundance of available foods [11], [12]. In this latter case, an animal should forage for foods having a nutrient content that best matches its immediate multiple nutritional demands. Currently, much of the literature on seasonal shifts in animal foraging behavior is descriptive, relating the spatiotemporal relationship between animals and their foods (e.g., [13], [14]), and using food preference as an indicator of the immediate requirement for nutrients contained in exploited foods [15], [16]. To our knowledge, no studies have attempted to experimentally demonstrate how seasonality *per se* modifies foraging behaviors associated with nutrient regulation.

For a broad range of reasons it is challenging to study the effects of seasonality on the shifts in foraging behavior of vertebrates, particularly as it relates to nutrient regulation. In contrast, colonies of social insects provide an excellent, experimentally tractable model. First, social insect colonies are a long-lived ‘superorganism’ [17] that experience multiple yearly cycles of seasonal changes in both food availability and demand. Although reproductive queens are typically the only colony member that directly experience such yearly cycles, remarkably queens of some social insects can live >10 years [18]. But even if the founding queen dies, replacement by related young queens can allow colonies of some ant species to persist for multiple decades [19]. Second, colonies of social insects have been shown from both laboratory and field studies to actively regulate nutrient intake [20], [21], [22]. Finally, insect and non-insect societies parallel one another in many aspects [17]. Among these is the requirement to obtain enough nutrients, and in the correct ratios, to promote the collective well-being of the society. Although individuals of social insect colonies differ in task and nutritional requirements, individuals work to promote the survival of the whole society, perhaps even beyond their own lifetime. In the short term, intricate interactions between adult and developing nestmates are believed to direct food collection behaviors of foragers. Whether a mechanism also exists that directs food collection behavior of foragers for long-term colony well-being, and the extent to which it is dynamic and can be modified in response to different nutritional and environmental conditions, remains poorly understood.

In this study we used the red-imported fire ant, *Solenopsis invicta* to examine how seasonality *per se* affects foraging behavior. We conducted two experiments that utilized summer- and fall-collected ant colonies, split into experimental colonies with similar demographic traits (a single queen, plus similar numbers of workers and brood). In our first experiment we restricted summer- and fall-colonies to foods with fixed protein (p) to carbohydrate (c) ratios. In our second experiment, summer- and fall-collected colonies were allowed to self-select their protein-carbohydrate intake. For both experiments we measured and compared the amount of protein and carbohydrate collected, and ingested, by summer- and fall-collected colonies. All experiments were conducted over a 5-week period, in a growth chamber set with fixed photoperiod, temperature and humidity levels. We show that summer- and fall-collected ants practice different protein-carbohydrate regulation strategies, and discuss the functional significance of this contrasting behavior in terms of the value of protein and carbohydrates for ant colonies.

## Results

### No-Choice Experiment

**Figure 1:**
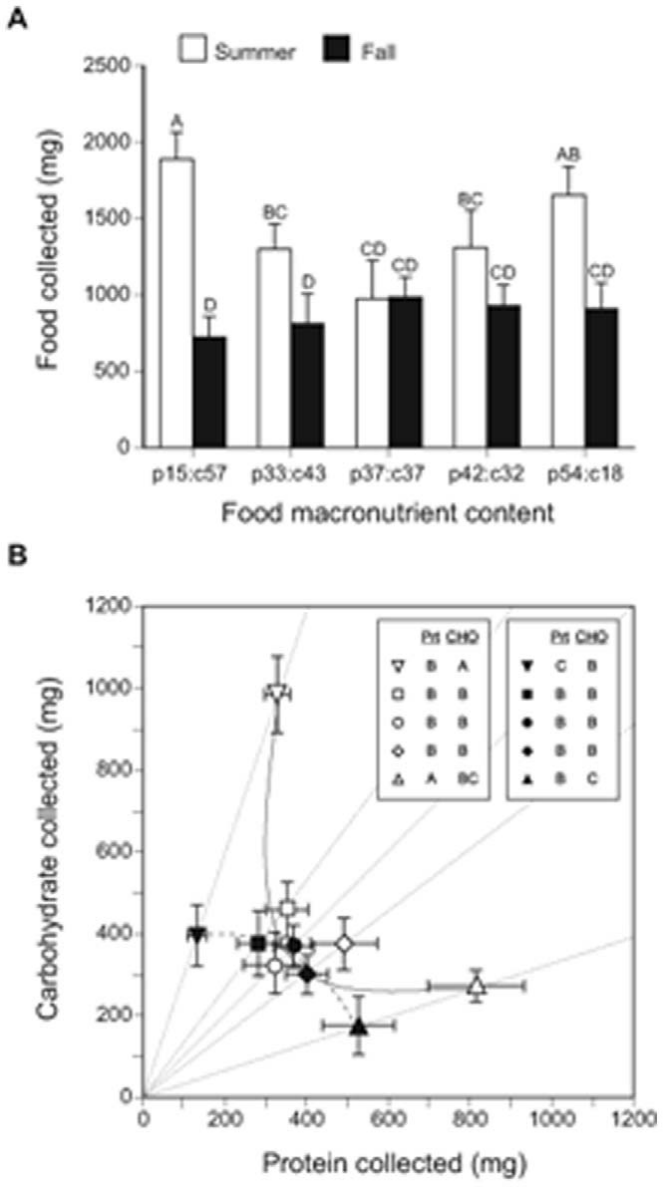
Food and nutrient collection for summer and fall colonies on diets with different protein-carbohydrate ratios. Panel (A) shows the mean (± S.E.) amount of food collected, over 5 weeks, by summer (open columns) and fall (filled columns) colonies. Significant differences in food collection between the food treatments for summer colonies are indicated by different capital letters above columns. Panel (B) shows the mean (± S.E.) amount of protein and carbohydrate collected, for the same five diets, for summer (open symbols) and fall (closed symbols) colonies. The lines emanating from the origin represent the p:c ratio of five diets: inverted triangle (19% protein, 57% carbohydrate; p19:c57); square (p33:c43); circle (p37:c37); diamond (p42:c32); and triangle (p54:c18). The solid and long-dashed curves represent the fitted intake arrays (see text for details) for summer and fall colonies, respectively. Different letters in the figure legend indicates significant differences in protein and carbohydrate collection between the diets (for each season).

In this experiment summer and fall colonies were restricted to feeding on one of five singly-available foods with different protein (p) to carbohydrate (c) ratios (see Materials and Methods and Table S1). Summer colonies collected more food, on average, than fall colonies, but more interestingly, a significant diet-by-season interaction was detected (Fig. 1A; Table 1). Summer and fall colonies collected similar amounts of balanced (37% protein, 37% digestible carbohydrate (p37:c37)) and slightly protein-biased foods (p42:c32), but summer colonies collected significantly more food when it was slightly carbohydrate-biased (p33:c43). The differences in food collection between summer and fall colonies were even more pronounced on the two highly unbalanced foods (p19:c57 and p54:c18).

**Table 1:** Results from ANOVA and MANOVA on food collection and consumption in no-choice experiments.

No-choice	Source	*F*	df	*P*
Total food collected	Season	26.63	1,57	<0.001
	Food	1.31	4,54	0.278
	Season-by-Food	3.12	4,54	0.023
Protein collected*	Season	10.23	1,57	0.002
	Food	8.58	4,54	<0.001
	Season-by-Food	2.37	4,54	0.065
Carbohydrate collected*	Season	9.57	1,57	0.003
	Food	7.71	4,54	<0.001
	Season-by-Food	2.34	4,54	0.690
Protein and carbohydrate	Season	0.55	2,46	0.580
consumed*	Food	10.29	8,92	<0.001
	Season-by-Food	2.88	8,92	0.007

These analyses tested the effect of season and dietary factors on total food and macronutrient collection, and macronutrient consumption by summer and fall colonies on no-choice foods.

*Analyses conducted on log-transformed data.

Nutrient collection, expressed as the amount of protein and carbohydrate gathered, is shown in Figure 1B. There was a significant season and food effect for both protein and carbohydrate collection, and for protein there was a marginally significant season-by-food interaction (Table 1). When a ‘collection array’ (*sensu* [23]) was fit separately to the summer and fall protein-carbohydrate collection points, to explore whether summer and fall colonies differed in their nutrient regulation strategies (reviewed by [24]), a striking difference was observed (Fig. 1B). A strongly concave intake array was observed for summer colonies, but for the fall colonies a convex intake array was observed. The most noticeable difference is seen on the two most extreme foods (p19:c57 and p54:c18). On these two diets, summer colonies ate significantly greater combined amounts of protein and carbohydrate than did fall colonies. The implication of these different nutrient regulation strategies is considered in the discussion.

**Figure 2:**
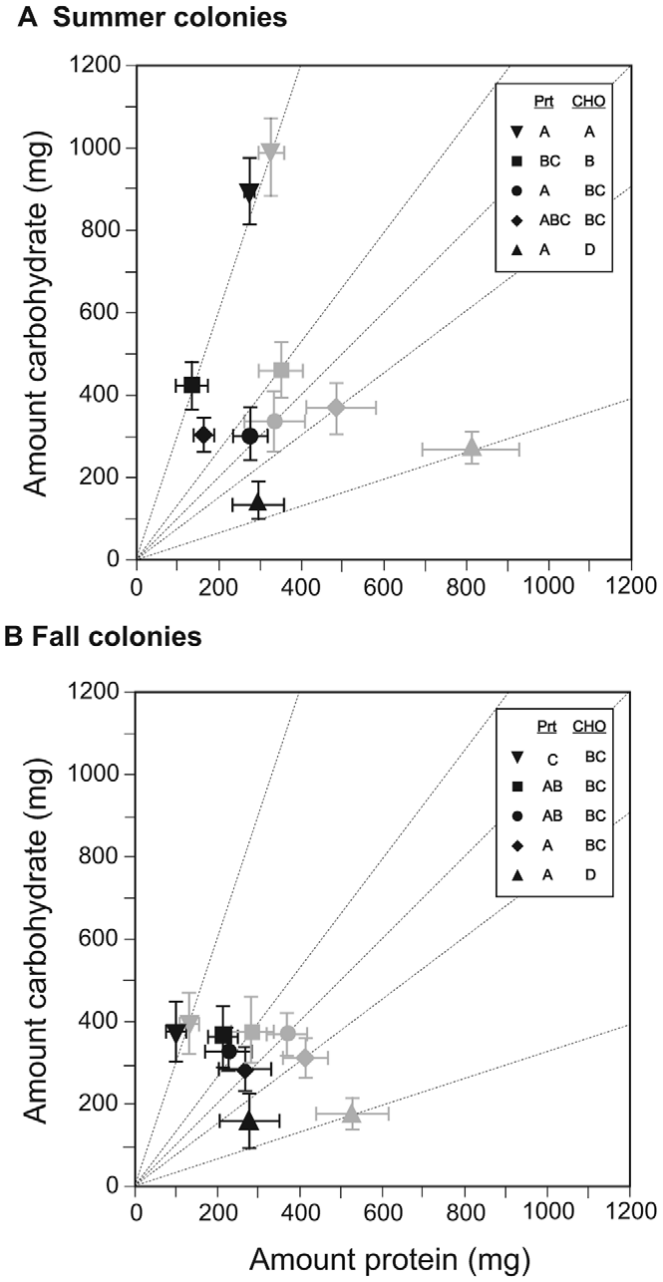
Nutrient consumption for summer and fall colonies on diets with different protein-carbohydrate ratios. These figures show the mean (± S.E.) total amount of protein and carbohydrate consumed [dark symbols in both panel (A) and (B)] and collected [light grey symbols in both panel (A) and (B); data from Figure 1B]. The dashed lines emanating from the origin represent the protein-carbohydrate (p:c) ratio of five diets: inverted triangle (19% protein, 57% carbohydrate; p19:c57); square (p33:c43); circle (p37:c37); diamond (p42:c32); and triangle (p54:c18). Protein consumption was calculated by subtracting the amount of protein in unconsumed foods from the amount of protein from collected food; carbohydrate consumption was calculated using a similar approach (see Supporting Methods and Results for complete details; also see Fig. S1). Different letters in the figure legend indicate significant differences in protein and carbohydrate consumption between the diets.

An important observation in this experiment was that ants did not eat all the food they collected; the majority of summer and fall colonies contained some unconsumed food (see Supporting Methods and Results; Figure S1). Interestingly, chemical analyses of unconsumed foods revealed a different protein-carbohydrate profile compared to the respective experimental food (Table S2). In general, very little carbohydrate was detected in any of the unconsumed foods (see Supporting Methods and Results), indicating that summer and fall ants consumed most of the carbohydrate they collected (Fig 2A & 2B). In contrast, the protein content of the cached food increased proportionately with the protein content of the food (Fig. 2A & 2B).

After taking into account the amount of both protein and carbohydrate contained in unconsumed foods, actual protein and carbohydrate consumption was compared. This revealed a significant season-by-diet interaction (Fig. 2A & 2B; Table 1), which was investigated more thoroughly by conducting separate *post hoc* analyses for protein and carbohydrate consumption. Here, and between seasons, the only difference in protein-carbohydrate consumption was on the highly carbohydrate-biased treatment (p19:c57). Summer colonies on this treatment, compared to fall colonies, consumed significantly greater amounts of both protein and carbohydrate (Fig. 2A & 2B).

**Figure 3:**
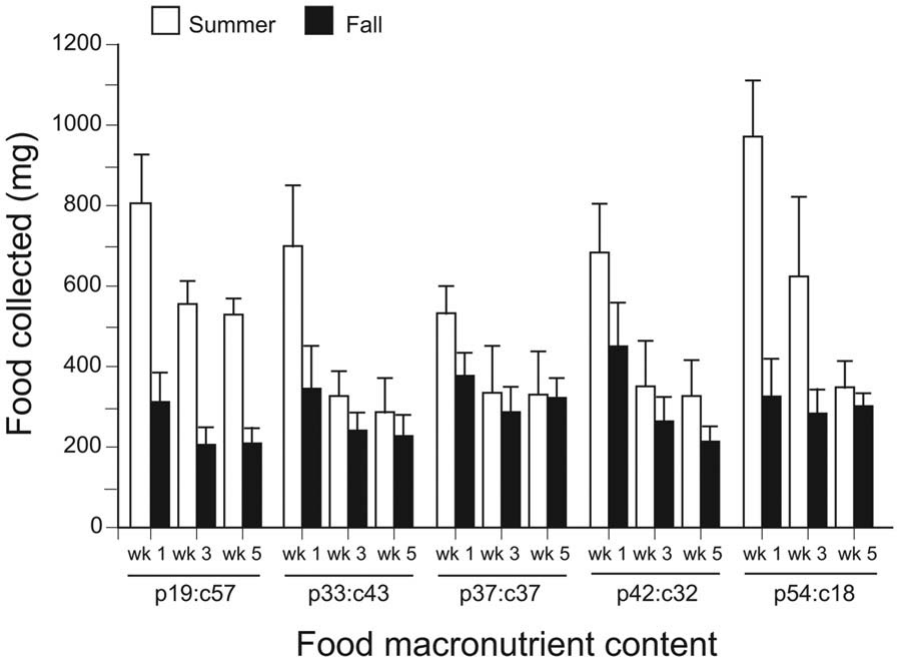
Five-week food collection patterns for summer and fall colonies on diets with different protein-carbohydrate ratios. Bars represent the mean (± S.E.) total amount of food collected (for weeks 1, 3, and 5) on each diet for summer (open columns) and fall (filled columns) colonies.

Figure 3 shows the weekly pattern of food collection by summer and fall colonies. Generally, the amount of food collected was greatest during week one and decreased successively in weeks three and five (repeated measures ANOVA of log-transformed data; *F*_2, 47_ = 19.62, *P*<0.001). More importantly, however, a significant season-by-time interaction was observed (*F*_2, 47_ = 4.96, *P* = 0.011). Here food collection was higher for summer colonies in week one, but by week five the amount of food collected by summer and fall colonies was similar (Fig. 3). No diet-by-time (*F*_8, 94_ = 0.86, *P* = 0.550) or season-by-diet-by-time (*F*_8, 94_ = 0.96, *P* = 0.471) interaction was observed.

### Choice Experiment

In this experiment, colonies were presented with paired nutritionally complimentary foods (three possible combinations), allowing colonies to self-select their protein and carbohydrate intake. Two key results were obtained. First, neither summer or fall foragers fed randomly (Table S3); on all food pairings colonies always showed a preference (in terms of the total amount collected) for the carbohydrate-biased food (Fig. S2). Second, food collection patterns revealed that summer colonies amassed significantly more of each food than did fall colonies, except for colonies on treatments that paired the p33:c43 and p54:c18 foods (Table 2, Fig. S3).

**Table 2:** Results from ANOVA and MANOVA on food collection and consumption in choice experiments.

Choice	Source	*F*	df	*P*
Total food collected	Season	13.13	1,34	0.001
	Food Pairing	4.34	2,33	0.023
	Season-by-Food Pairing	2.13	2,33	0.137
Protein and carbohydrate	Season	3.46	2,29	0.045
collected	Food Pairing	19.50	4,58	<0.001
	Season-by-Food Pairing	1.34	4,58	0.265
Protein and carbohydrate	Season	3.10	1,28	0.061
consumed	Food Pairing	5.90	4,56	<0.001
	Season-by-Food Pairing	1.41	4,56	0.242

These analyses tested the effect of season and dietary factors on total food and macronutrient collection, and macronutrient consumption by summer and fall colonies on choice food pairings.

**Figure 4:**
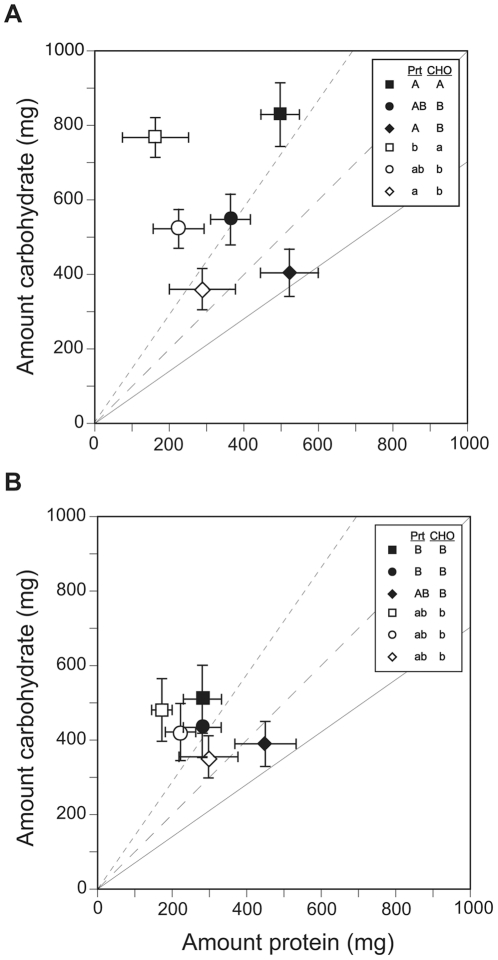
Nutrient collection and consumption for summer and fall colonies on nutritionally complimentary food pairings. The mean (± S.E.) total amounts of protein and carbohydrate collected (filled symbols) and consumed (open symbols) by summer (A) and fall (B) colonies on the three food pairings comprising choice treatments over five weeks. Symbols represent food pairings: Squares (treatment 1)  = food p19:c57 with food p42:c32; Circles (treatment 2)  =  food p19:c57 with food p54:c18; and Diamonds (treatment 3)  =  food p33:c43 with food p54:c18. Small dash, large dash, and solid lines emanating from the origin represent the protein:carbohydrate ratio of collected food if foragers collected equally from the two food dishes comprising treatment one, two, and three, respectively. Different capital and lower-case letters in the figure legend indicates significant differences in protein and carbohydrate collection and consumption, respectively between the diets (for each season).

Food collection expressed in terms of the amount of protein and carbohydrate is shown in Figure 4A and 4B. Protein and carbohydrate collection were both significantly affected by season and food pairing, but no season-by-food pairing interaction was observed (Table 2). In general, summer colonies collected greater combined amounts of protein and carbohydrates. With respect to comparisons between the three food pairings, carbohydrate collection was similar on treatments that had a dish of highly biased-protein (p54:c18), but significantly higher on the treatment lacking the highly biased-protein food. Significant differences in protein collection were also observed between the three food pairings. However, the difference in carbohydrate collection between treatments was greater than the difference in protein collection between treatments (Fig. 4A & 4B).

As in no-choice experiments, the majority of ants did not eat all the food they collected (see Supporting Methods and Results), and seasonal colonies on most treatments (except fall colonies feeding on the protein-biased food pairing) extracted much of the carbohydrate from collected foods (Table S4). Therefore, actual protein and carbohydrate consumption (as opposed to collection) was compared across the three food pairings after taking into account the amount of both protein and carbohydrate contained in unconsumed foods (see Supporting Methods and Results). This analysis revealed a marginally significant difference between seasons, but a highly significant effect of food pairing on protein and carbohydrate consumption (Figs. 4A and 4B; Table 2). This outcome was investigated further by conducting separate *post hoc* analyses for both protein and carbohydrate consumption. Summer and fall colonies consumed very similar amounts of protein, but summer colonies feeding on the treatment pairing foods p19:c57 and p42:c32 consumed more carbohydrate than colonies on the other treatments (Fig. 4A).

The weekly pattern of total food collection for summer and fall colonies on choice food pairings is shown in Fig. S3. Food collection was greatest during week one and decreased successively in weeks three and five (repeated measures ANOVA of log-transformed data; *F*_2, 29_ = 14.18, *P*<0.001), and a significant season-by-time effect was observed (*F*_2, 29_ = 3.89, *P* = 0.032). Summer colonies collected, on average, greater amounts of food in week one, but at the end of the experiment (week 5), summer and fall colonies were collecting similar amounts of food. No treatment-by-time interaction (*F*_4, 58_ = 2.09, *P* = 0.093), or season-by-treatment-by-time interaction (*F*_4, 58_ = 1.27, *P* = 0.291) related to food collection was observed.

## Discussion

Insect societies differ from long-lived solitary animals in many respects, but both share in common the ability to regulate their nutrient intake [20], [21], [24], [25], and both experience regular cyclical shifts in environmental conditions (e.g., see Figure S4A and S4B). In this study we show that the nutrient content of available foods can influence food collection behavior in fire ants, but that the nutrient regulation strategies employed by fire ants differ dramatically between the summer and fall. Importantly, our experimental set-up (demographically similar experimental colonies, *ad libitum* feeding conditions, plus fixed temperature, photoperiod and humidity levels) reveals that these contrasting nutrient regulation strategies appear pre-programmed, and are independent of colony composition, food availability and environmental conditions. Previous studies have shown that the response of animals to the nutrient content of foods is dynamic, and can change depending on an animal's developmental, reproductive, and/or energetic demands (reviewed in [23], [24]). Our results are novel because they demonstrate that this nutrient regulation in animals is also seasonally dynamic.

Our choice experiments, as well as other studies exploring nutrient regulation in ants [20], [21], [22], [25], demonstrate that ants prefer a balanced, to slightly carbohydrate-biased diet. Dussutour and Simpson [20] showed the functional significance of a carbohydrate-biased diet for ants – increased worker and larval survival relative to feeding on a protein-biased diet. We too have found similar results with fire ants [21]. However, our current experiments indicate that summer and fall ants practice seasonally distinct foraging behaviors with respect to regulation of nutrient intake. The best way to understand this contrasting behavior is to focus on results from the no-choice experiments, specifically the protein and carbohydrate collection and consumption patterns on the two most unbalanced foods (p19:c57 and p54:c18), and to consider the functional value of these two key macronutrients. Protein provides amino acids that are used predominately by larvae to grow (and by extension the colony), while carbohydrates (e.g., sugars) are used as a substrate for energy. On the strongly carbohydrate-biased diets (p19:c57), protein is limited relative to carbohydrates, so to collect sufficient quantities of protein for larval growth, large quantities of this food would need to have been collected. Ants showed this compensatory behavior in the summer, but not in the fall. With respect to carbohydrates, Dussutour and Simpson [25] have shown ants strongly regulate carbohydrate, and do so under a number of different conditions. In our no-choice experiment, carbohydrates were most limited on the strongly protein-biased diet (p54:c18), so here ants would need to have collected large quantities of food to fuel their energy demands. Summer ants, but not fall ants, showed this compensatory food collecting behavior. Our experimental setup (food supply, colony demographics, photoperiod, temperature, humidity) was identical for both summer and fall colonies on these two highly imbalanced foods, so our results suggest there is a season-specific cue directing them to display such contrasting nutrient regulation behavior.

Despite differences in food collection, both summer and fall colonies regulated their protein intake to similar levels through manipulation of the nutrient content of their food. A strong, directed protein regulation response is not surprising considering ants maintain at least some brood throughout the year (authors' observations, [26], [27], [28]); there is a constant demand for protein. Recently Dussutour and Simpson [20] documented the strong role that larvae play in protein regulation behavior; ant colonies that lack brood prefer carbohydrate-biased diets, while those with brood prefer a more balanced protein-carbohydrate intake. However, Dussutour and Simpson [20] also showed that too much protein can be toxic for ants, so regulating protein intake to a fixed level is a mechanism for keeping the entire colony healthy. With respect to carbohydrate regulation, ants from our experiments always consumed most of the carbohydrate they collected. Carbohydrates, in contrast to protein, are equally valuable for both workers and larvae. In workers, carbohydrates fuel foraging activities, and can be used to build lipid reserves, and in larvae they enhance development when matched with dietary protein [29]. That ants should efficiently use the carbohydrates they collect makes sense given its broad value to members of a colony, but the extent to which ants are willing to process excess protein-biased foods to increase their carbohydrate intake is likely limited by the toxic effects associated with eating too much protein [20].

In the choice experiments, similar patterns in nutrient collection and consumption are also evident for summer and fall colonies. Summer colonies collected excess protein, but both summer and fall colonies both regulated protein consumption to similar levels. In contrast, summer colonies, but not fall colonies, increased their carbohydrate consumption when more carbohydrate-rich foods were available in the environment (e.g., pairing food p19:c57 with p42:c32). Previous studies suggest that some mammals (reviewed in [30]) and birds [31] naturally exhibit seasonal differences in food collection behavior, and also under *ad libitum* food availability [30]. For example, North American ungulate species reduce food intake during winter months, and Parker *et al*. [30] suggests that this behavior is a strategy to avoid physiological costs (i.e., due to reduced metabolic functions) associated with consuming excess food. However, one advantage social insects may have over many solitary animals is that not all collected food is immediately consumed; excess nutrients can be stored for later use. Hoarding food by ants is thought to be a common phenomenon, and is even referred to in ancient writings (Proverbs 6: 6–8, and Aesop's fable, The Ant and the Grasshopper). However, other than anecdotal evidence, little is actually known about hoarding behavior in ants. Some ants hoard liquid carbohydrate (e.g., remarkable storage capacity for liquids of replete castes of ‘honey pot’ ants (*Myrmecocystus* spp. [32]), but only recently has experimental evidence shown that excess collected protein is stored inside colonies for possible later use [33]. Colonies may utilize hoarded protein to rear a winter batch of larvae, particularly larvae of reproductive castes [33]. Not unlike other animals that collect and hoard excess foods (e.g., squirrels and pika), ants may be collecting excess protein when abundant for use when this nutrient is scarce (or for development of larger batches of brood that are normally found in summer colonies in the field [26]), but a cue other than current food abundance and demand appear to direct collection of excess protein in ants. We suggest this latter point reflects a programmed priority of summer colonies to collect protein in amounts above that funding colony growth. Seasonal shifts in hoarding behavior also occurs in other animals, and this behavior has been linked to photoperiod; increasing day-length decreases hoarding behavior in hamsters, deermice, and chickadees [34], [35], [36].

Based on the temporal feeding patterns in both the no-choice experiments, and the consistency of our experimental regime for both summer- and fall-collected ants, we suggest that photoperiod is also a potential cue directing contrasting seasonal foraging strategies of ants. In the first week of feeding, summer colonies across all diets consistently collected more food than did fall colonies. However, food collection in the summer colonies consistently declined over the course of the experiment, and at the last week of the experiment collection amounts for summer and fall colonies were similar on all diets except the strongly carbohydrate-biased one (p19:c57); for this food, collection remained relatively high). In our experiments the natural photoperiod experienced by summer source colonies was (light:dark) 14hr:10hr, while that experienced by fall source colonies was (11.5hr:12.5hr). However, the experimental photoperiod we used (12hr:12hr) more closely matched the natural photoperiod experienced by fall colonies. Thus, summer colonies, in contrast to fall colonies, experienced a decrease in day-length. Changing photoperiod has been shown previously to affect several aspects of animal food collection behavior. For example, increased experimental photoperiod prolonged the length of nocturnal foraging in the polychaete *Nereis virens* [37], prolonged foraging bouts in Siberian hamsters [16], and dampened nocturnal foraging intensity in a grain beetle [38]. Temperature is another environmental factor that might affect foraging behavior, and our summer and fall colonies did experience different natural temperature regimes, in terms of absolute temperatures. The periodicity of certain animal behaviors can become entrained to a thermal cue (see [39]), and the current metabolic status of some animals can be influenced by an experienced thermal history [40]. However, the degree to which either temperature or photoperiod affects nutrient regulation strategies, or whether they interact to produce the striking differences we observed in our study, has yet to be explored experimentally.

Seasonal shifts in food collection behavior, as they relate to seasonal adaptations in animals, have received surprisingly little attention in the literature [41], [42]. Our study demonstrates, for the first time, a link between seasonal food collection behavior and nutrient regulation strategies. Employing season-specific nutrient regulation strategies may be an adaptation of many animals to meet current and long-term nutrient demands when nutrient-rich foods are abundant, to conserve energy when such foods are less abundant, and to avoid instances of food stress associated with ingestion of nutrients beyond current physiological constraints [43]. Understanding seasonal shifts in animal food collection behavior based on contrasting nutrient regulatory strategies may have far-reaching ecological importance, including providing a predictive model of seasonal patterns in animal food collection behavior based on the relative nutrient content of available foods.

## Materials and Methods

### Experimental ant colonies and laboratory conditions

Polygynous colonies were collected between July 15 and July 25, 2009 and between October 20 and October 31, 2009, from the Riverside campus of Texas A&M University, USA. Monogynous experimental colonies were formed from each of the source colonies. Each experimental colony consisted of a single wingless queen, 1000 mg workers (haphazardly chosen from both nesting and foraging areas), 200 mg larvae and 100 mg pupae (the latter forms not of future reproductive castes). Eight experimental colonies were formed from each source colony and allocated to each of the treatments (see below). In cases when less than eight experimental colonies were formed from a single source colony, experimental colonies were randomly assigned to an experimental treatment. Six replicate colonies were assigned to each of the treatments. Experimental colonies were each housed in a 24.6 cm×19.2 cm×9.5 cm plastic box, and provided as a nest chamber a 15 cm diameter lidded and covered Petri dish, filled approximately half-full with hardened Castone® dental stone. Castone® substrate was moistened regularly to maintain a high humidity inside nest chambers [44]. Colonies were provided an *ad libitum* water source. Colonies were housed in an insectary and exposed to a 12h:12h L:D diel cycle under fluorescent lighting, and maintained at 26°C temperature and at ambient humidity (45–60%). For East Texas, in June the natural photoperiod is 14h:10h L:D, and in October the photoperiod is 11h:13h L:D. For this study, experimental summer colonies were exposed to a photoperiod having a significantly shortened period of light than summer field colonies. In contrast, experimental fall colonies were exposed to a more natural photoperiod.

### Experimental Foods

Experimental diets consisted of five agar-based synthetic foods created by combining methods of Cook *et al*., [21], Dussutour & Simpon [45], and Stratka & Feldhaar [46] and ranged in total protein (p) and carbohydrate (c) content from 79–83% (see Table S1). The five diets, expressed as the percentage of diet total dry mass, were: (1) p54:c18, (2) p42:c32, (3) p37:c37, (4) p33:c43, and (5) p19:c57. The dietary protein component consisted mainly of an approximate 1∶1 mixture of whey protein concentrate and calcium caseinate. There was an additional protein source from whole-egg powder, the amount and proportion of which remained constant across diets. Nearly half of the whole egg powder consisted of lipids (fats and sterols). The dietary carbohydrate used was sucrose.

### Experimental protocol

Both no-choice and choice experiments were run concurrently for each season. No-choice experiments consisted of five dietary treatments having a protein-to-carbohydrate ratio ranging from 0.3 to 3.0: Treatment 1 (p19:c57), Treatment 2 (p33:c43), Treatment 3 (p37:c37), Treatment 4 (p42:c32), and Treatment 5 (p54:c18). Choice experiments consisted of three dietary treatments each composed of a pairing of nutritionally complimentary experimental foods: Treatment 1 (p42:c32 and p19:c57), Treatment 2 (p54:c18 and p19:c57), and Treatment 3 (p33:c43 and p54:c18). Colonies were provided with fresh food every day for five weeks. One cm^3^ piece of each of the experimental foods was placed in a small, pre-weighed plastic weighing boat, weighed to 0.01 mg, and then placed in each of the experimental colonies. Three replicate, preweighed weighing boats containing each of the five foods was placed in different areas of the insectary, and acted as controls for evaporative water loss. After 24 hours, all food dishes, including controls, were collected and placed in a 35°C drying oven for ∼48 hours. Once thoroughly dried, pre-weighed weighing dishes containing remaining food, were each re-weighed to obtain the dry weight. The amount of food collected (as dry weight) by colonies was obtained by first, generating regression plots of wet- and dry-weights of control foods, then using the equation of the linear function corresponding to the best fit to these data, computing the evaporative weight lost for each experimental food. The difference between this value and final dry weights of experimental foods gave the amount of each food that was collected by colonies each day. The majority of both summer and fall colonies did not consume all the food collected; many cached and/or discarded unconsumed foods. The degree to which colonies manipulated the protein and carbohydrate content of collected foods was determined by using the Bradford and phenol-sulfuric acid assays, respectively, to determine the protein and carbohydrate content of unconsumed foods (see Supporting Methods and Results).

### Statistical analyses

Parametric statistics were used to conduct all analyses. Prior to analysis, data were checked for normality and for equal variances, using the Shapiro-Wilk test and O'Brien test, respectively. If data did not meet these criteria, data were log-transformed (signified in text). Analyses of total food (and protein and carbohydrate) collection in no-choice experiments were conducted using ANOVA. Analyses of total food collection in choice experiments were conducted using MANOVA; foragers could independently collect either of the two paired nutritionally complimentary foods. For choice experiments, separate *t*-tests were conducted to determine whether workers foraged selectively between the two foods of each food pairing. These tests compared the p:c ratios (total protein and carbohydrate content) of each food pairing to the p:c ratio of the foods actually collected (total protein and carbohydrate content). Analyses of protein and carbohydrate collection were conducted using ANOVA; experimental foods contained both protein and carbohydrate, and thus collection of protein and carbohydrate was not independent. Analyses of food consumption in both no-choice and choice experiments were conducted using MANOVA; ants can selectively extract protein or carbohydrate from collected foods [20]. Weekly total food collection was analyzed using repeated measure two-factor ANOVA. Following analyses, and where applicable, least-square means Student's *post hoc* tests were conducted from results generated from univariate ANOVA. All analyses were conducted using the software package Jump 7.02 (SAS Institute, Inc.).

## Supporting Information

Figure S1 **Amounts of unconsumed food for summer and fall colonies on foods with different protein-carbohydrate ratios.** Mean (± S.E.) total amount of unconsumed foods from summer (open columns) and fall (filled columns) colonies caching excess food on the five no-choice treatments (A), and the mean (± S.E.) proportion of total collected food that remained unconsumed (B). Different upper case letters above columns represent significant within-season and across treatment differences from Student's *post hoc* tests (*P*<0.05) for summer and fall colonies.
(TIF)

Figure S2 **Food collection for summer and fall colonies on nutritionally complimentary food pairings.** Mean (+S.E.) total amount of food collected from each of the two foods comprising the three dietary choice treatments (A–C) over five weeks by summer and fall colonies. Bars are shaded to correspond with each of the four experimental foods expressed as the percent protein and carbohydrate content: white bars  =  food p19:c57, light grey bars  =  food p33:c43, dark grey bars  =  food p42:c32, and black bars  =  food p54:c18. Different capital letters above columns represent significant differences from Student's *post-hoc* tests (*P*<0.05) comparing collection of foods one and two, respectively.
(TIF)

Figure S3 **Five-week food collection patterns for summer and fall colonies on nutritionally complimentary food pairings.** Mean (± S.E.) weekly total amount of food collected by summer (open columns) and fall (filled columns) colonies feeding on food pairings comprising choice experiments.
(TIF)

Figure S4 **Environmental conditions at source colony collection site.** Summer (A) and fall (B) in Brazos County, Texas, USA, near location where source colonies of *Solenopsis invicta* were collected.
(TIF)

Table S1 **Dietary components of experimental foods used in both choice and no-choice treatments**. Amounts are based on 60 g total dry weight. The amounts of both proteins and sucrose used are after subtracting product impurities based on product nutritional labels (see [21] for details).
(DOC)

Table S2 **Results from one-tailed t-tests examining the manipulation of collected foods by summer and fall colonies on no-choice treatments.** The mean p:c ratio of unconsumed foods is compared to that of each experimental food. We assumed *a priori* that colonies would selectively extract carbohydrate over protein from collected foods [20], [21]. Analysis was conducted on log-transformed data for fall colonies feeding on food p19:c57.
(DOC)

Table S3 **Results of two-tailed** ***t*** **-tests analyzing worker selectivity between the two foods comprising the three choice treatments.** The mean p:c ratio of total food collected by summer and fall colonies on choice treatments was compared to the mean p:c ratio of foods comprising choice treatments. Significant *P*-value (α = 0.05) indicates selective foraging (i.e., non-random collection) between the two foods.
(DOC)

Table S4 **Results from one-tailed t-tests examining the manipulation of collected foods by summer and fall colonies on dietary choice treatments.** The mean p:c ratio (± s.e.m) of unconsumed foods is compared to that of total combined collected foods. We assumed *a priori* that colonies would selectively extract carbohydrate over protein from collected foods [20], [21]. Analysis was conducted on log-transformed data for summer colonies feeding on food pairing p54:c18 & p33:c43.
(DOC)

Supporting Methods and Results **Methods determining the nutrient content of unconsumed foods, and results of analyses revealing the degree to which ants manipulated the nutrient content of collected foods.**
(DOC)
