# Supplementary figures and images for: Seasonality Directs Contrasting Food Collection Behavior and Nutrient Regulation Strategies in Ants
Source: PLoS One. 2011 Sep 26;6(9):e25407. doi: 10.1371/journal.pone.0025407 (PMC3180453; doi:10.1371/journal.pone.0025407)

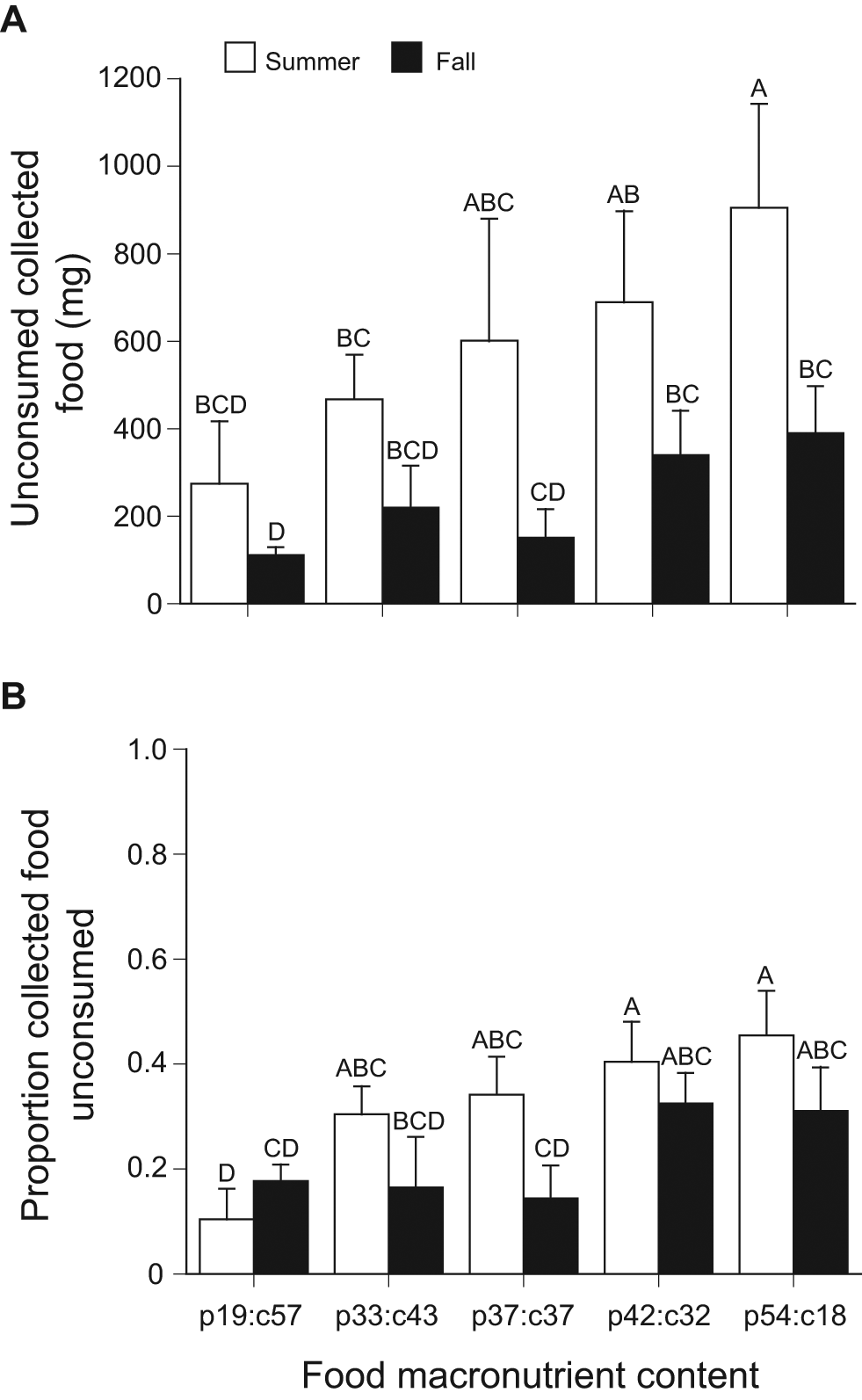

Supplement: Figure S1 — Amounts of unconsumed food for summer and fall colonies on foods with different protein-carbohydrate ratios. Mean (± S.E.) total amount of unconsumed foods from summer (open columns) and fall (filled columns) colonies caching excess food on the five no-choice treatments (A), and the mean (± S.E.) proportion of total collected food that remained unconsumed (B). Different upper case letters above columns represent significant within-season and across treatment differences from Student's post hoc tests (P<0.05) for summer and fall colonies. (TIF) [file pone.0025407.s001.tif]

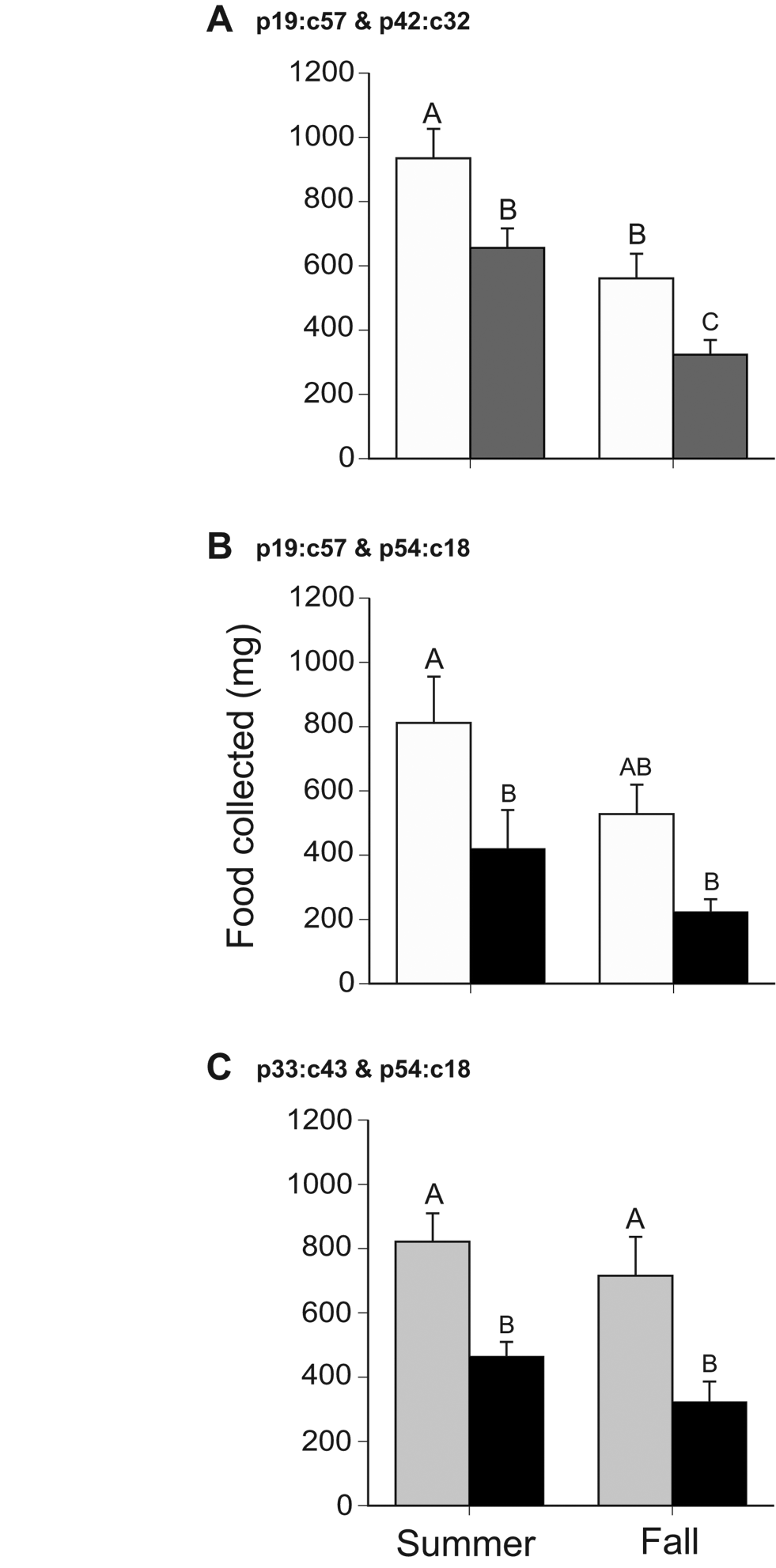

Supplement: Figure S2 — Food collection for summer and fall colonies on nutritionally complimentary food pairings. Mean (+S.E.) total amount of food collected from each of the two foods comprising the three dietary choice treatments (A–C) over five weeks by summer and fall colonies. Bars are shaded to correspond with each of the four experimental foods expressed as the percent protein and carbohydrate content: white bars = food p19:c57, light grey bars = food p33:c43, dark grey bars = food p42:c32, and black bars = food p54:c18. Different capital letters above columns represent significant differences from Student's post-hoc tests (P<0.05) comparing collection of foods one and two, respectively. (TIF) [file pone.0025407.s002.tif]

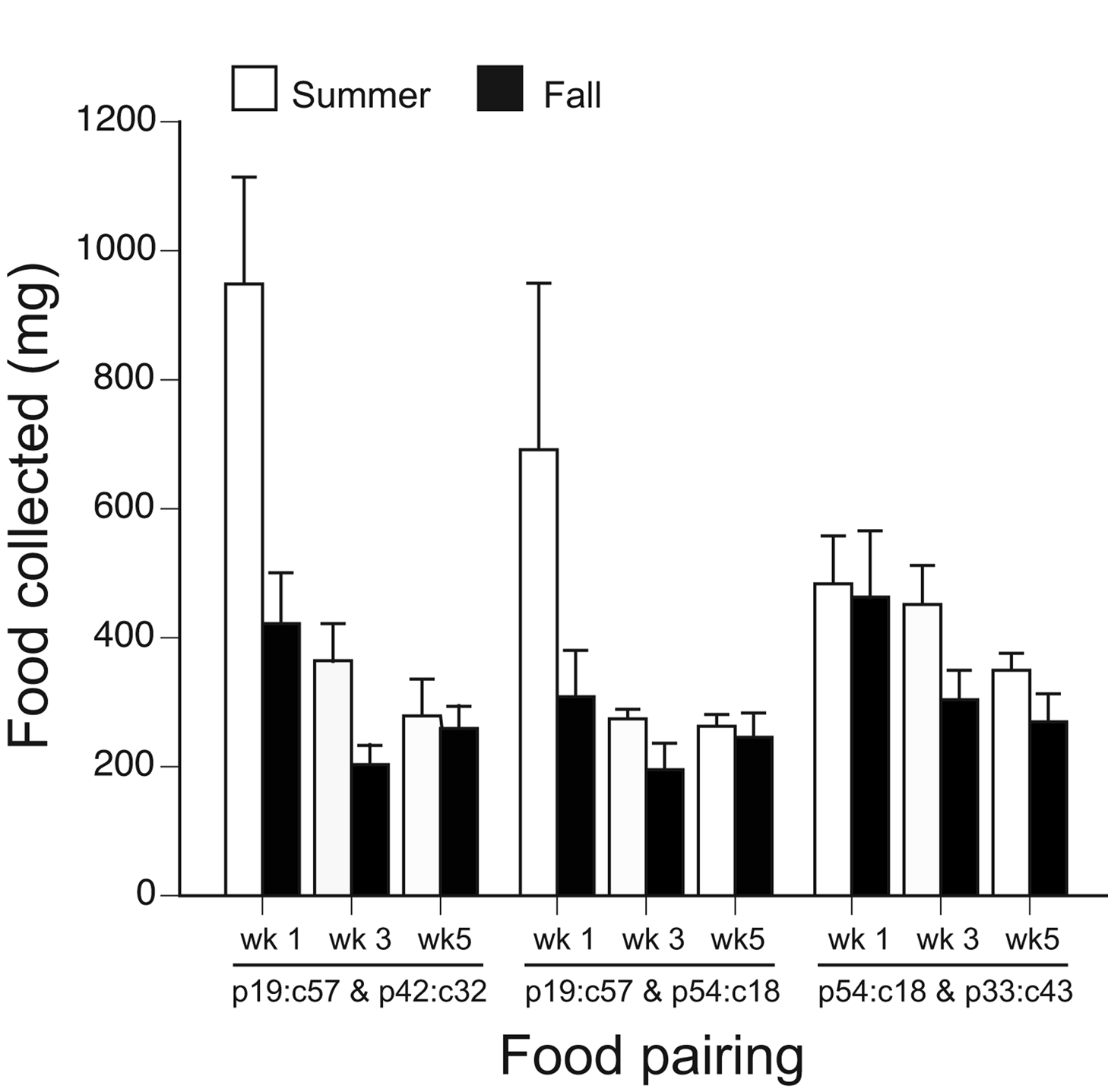

Supplement: Figure S3 — Five-week food collection patterns for summer and fall colonies on nutritionally complimentary food pairings. Mean (± S.E.) weekly total amount of food collected by summer (open columns) and fall (filled columns) colonies feeding on food pairings comprising choice experiments. (TIF) [file pone.0025407.s003.tif]
