# Supplementary material for: Seasonality Directs Contrasting Food Collection Behavior and Nutrient Regulation Strategies in Ants
Source: PLoS One. 2011 Sep 26;6(9):e25407. doi: 10.1371/journal.pone.0025407 (PMC3180453; doi:10.1371/journal.pone.0025407)
Supplement: Table S1 — Dietary components of experimental foods used in both choice and no-choice treatments. Amounts are based on 60 g total dry weight. The amounts of both proteins and sucrose used are after subtracting product impurities based on product nutritional labels (see [21] for details). (DOC) [file pone.0025407.s005.doc]

|  | **Calcium** | **Whey** | **Egg** |  |  |  |  |  |
| --- | --- | --- | --- | --- | --- | --- | --- | --- |
| **Diet** | **caseinate** | **protein** | **powder** | **Sucrose** | **Salts** | **Vitamins** | **Agar** | **Water** |
|  | **(g)** | **(g)** | **(g)** | **(g)** | **(g)** | **(g)** | **(g)** | **(ml)** |
| p19:c57 | 3.9 | 4.3 | 15.6 | 49.1 | 1.5 | 0.4 | 4.0 | 300 |
| p33:c43 | 9.7 | 10.4 | 15.6 | 37.0 | 1.5 | 0.4 | 4.0 | 300 |
| p37:c37 | 12.1 | 13.4 | 15.6 | 32.7 | 1.5 | 0.4 | 5.0 | 300 |
| p42:c32 | 14.3 | 15.5 | 15.6 | 27.8 | 1.5 | 0.4 | 6.0 | 300 |
| p54:c18 | 20.3 | 22.4 | 15.6 | 16.4 | 1.5 | 0.4 | 6.0 | 300 |
