# Supplementary material for: Seasonality Directs Contrasting Food Collection Behavior and Nutrient Regulation Strategies in Ants
Source: PLoS One. 2011 Sep 26;6(9):e25407. doi: 10.1371/journal.pone.0025407 (PMC3180453; doi:10.1371/journal.pone.0025407)
Supplement: Table S2 — Results from one-tailed t-tests examining the manipulation of collected foods by summer and fall colonies on no-choice treatments. The mean p:c ratio of unconsumed foods is compared to that of each experimental food. We assumed a priori that colonies would selectively extract carbohydrate over protein from collected foods [20], [21]. Analysis was conducted on log-transformed data for fall colonies feeding on food p19:c57. (DOC) [file pone.0025407.s006.doc]

|  | **Experimental** | **p:c ratio of** |  |  |  |
| --- | --- | --- | --- | --- | --- |
| **Season** | **food** | **unconsumed food** | ***t-ratio*** | **df** | ***P > t*** |
| Summer |  |  |  |  |  |
|  | p19:c57 | 0.83 ± 0.30 | 1.65 | 3 | 0.098 |
|  | p33:c43 | 5.11 ± 1.51 | 2.89 | 5 | 0.022 |
|  | p37:c37 | 1.09 ± 0.27 | 0.35 | 5 | 0.372 |
|  | p42:c32 | 2.93 ± 0.56 | 2.89 | 5 | 0.017 |
|  | p54:c18 | 3.73 ± 0.69 | 1.07 | 5 | 0.167 |
| Fall |  |  |  |  |  |
|  | p19:c57 | 3.62 ± 2.22 | 1.89 | 5 | 0.058 |
|  | p33:c43 | 3.44 ± 1.17 | 2.3 | 3 | 0.052 |
|  | p37:c37 | 5.66 ± 2.31 | 2.02 | 3 | 0.051 |
|  | p42:c32 | 2.86 ± 0.69 | 2.21 | 5 | 0.039 |
|  | p54:c18 | 5.04 ± 0.96 | 2.13 | 4 | 0.05 |
|  |  |  |  |  |  |
