# Supplementary material for: Seasonality Directs Contrasting Food Collection Behavior and Nutrient Regulation Strategies in Ants
Source: PLoS One. 2011 Sep 26;6(9):e25407. doi: 10.1371/journal.pone.0025407 (PMC3180453; doi:10.1371/journal.pone.0025407)
Supplement: Table S3 — Results of two-tailed t -tests analyzing worker selectivity between the two foods comprising the three choice treatments. The mean p:c ratio of total food collected by summer and fall colonies on choice treatments was compared to the mean p:c ratio of foods comprising choice treatments. Significant P-value (α = 0.05) indicates selective foraging (i.e., non-random collection) between the two foods. (DOC) [file pone.0025407.s007.doc]

|  |  | **Expected** | **Actual** |  |  |
| --- | --- | --- | --- | --- | --- |
| **Treatment** | **Season** | **p:c ratio** | **p:c ratio** | ***t*-ratio** | ***P* > | *t* |** |
| p19:c57 & p42:c32 | Summer | 0.70 | 0.64 | -5.98 | 0.002 |
| p19:c57 & p54:c18 | Summer | 1.00 | 0.69 | -7.73 | 0.001 |
| p54:c18 & p33:c43 | Summer | 1.40 | 1.20 | -6.47 | 0.001 |
| p19:c57 & p42:c32 | Fall | 0.70 | 0.54 | -18.28 | <0.001 |
| p19:c57 & p54:c18 | Fall | 1.00 | 0.63 | -4.75 | 0.005 |
| p54:c18 & p33:c43 | Fall | 1.40 | 1.13 | -7.12 | 0.001 |
