# Supplementary material for: Seasonality Directs Contrasting Food Collection Behavior and Nutrient Regulation Strategies in Ants
Source: PLoS One. 2011 Sep 26;6(9):e25407. doi: 10.1371/journal.pone.0025407 (PMC3180453; doi:10.1371/journal.pone.0025407)
Supplement: Table S4 — Results from one-tailed t-tests examining the manipulation of collected foods by summer and fall colonies on dietary choice treatments. The mean p:c ratio (± s.e.m) of unconsumed foods is compared to that of total combined collected foods. We assumed a priori that colonies would selectively extract carbohydrate over protein from collected foods [20], [21]. Analysis was conducted on log-transformed data for summer colonies feeding on food pairing p54:c18 & p33:c43. (DOC) [file pone.0025407.s008.doc]

|  |  | **p:c ratio of** | **p:c ratio of** |  |  |
| --- | --- | --- | --- | --- | --- |
| **Treatment** | **Season** | **collected food** | **unconsumed food** | ***t*-ratio** | ***P* > *t*** |
| p19:c57 & p42:c32 | Summer | 0.55 ± 0.01 | 6.44 ± 0.63 | 9.40 | <0.001 |
| p19:c57 & p54:c18 | Summer | 0.67 ± 0.04 | 5.64 ± 0.71 | 6.95 | 0.003 |
| p54:c18 & p33:c43 | Summer | 1.35 ± 0.08 | 9.29 ± 3.02 | 6.05 | <0.001 |
| p19:c57 & p42:c32 | Fall | 0.55 ± 0.01 | 5.99 ± 1.57 | 3.47 | 0.009 |
| p19:c57 & p54:c18 | Fall | 0.69 ± 0.07 | 5.66 ± 0.96 | 5.16 | 0.003 |
| p54:c18 & p33:c43 | Fall | 1.16 ± 0.05 | 6.38 ± 1.38 | 3.79 | 0.010 |
