# Supplementary material for: Seasonality Directs Contrasting Food Collection Behavior and Nutrient Regulation Strategies in Ants
Source: PLoS One. 2011 Sep 26;6(9):e25407. doi: 10.1371/journal.pone.0025407 (PMC3180453; doi:10.1371/journal.pone.0025407)
Supplement: Supporting Methods and Results — Methods determining the nutrient content of unconsumed foods, and results of analyses revealing the degree to which ants manipulated the nutrient content of collected foods. (DOC) [file pone.0025407.s009.doc]

**Supporting Online Information**

**Cook, *et al*., Seasonality directs contrasting food collection behavior and nutrient regulation strategies in ants**

**Methods**

**Nutrient content of unconsumed foods.** Over the course of the experiment colonies did not consume all of the food collected, but rather cached excess food either inside or outside the nest. We conducted spectrophotometric assays to determine the protein and carbohydrate content of these unconsumed foods. First, unconsumed foods were collected at the end of the experiment, dried thoroughly, and weighed. After food was completely dried, samples were separately ground to a fine powder in liquid nitrogen and then re-dried. A sample of each of the experimental foods containing known quantities of protein and carbohydrate was treated as above and used as standards in assays (see below). Then, two 100 mg aliquots of each sample were placed separately into two 15 ml plastic centrifuge tubes (VWR International). To one tube (protein analysis) we added 10 ml tris-buffered saline (TBS) (20 mM Tris, 500 mM NaCl; pH = 7.5), and to the other (carbohydrate analysis) we added 10 ml of nano-pure de-ionized water. Samples were thoroughly mixed to dissolve miscible components (~ five minutes). A two-milliliter aliquot of each of the solutions was taken from each of the 15 ml tubes and placed in a 2-ml capped microcentrifuge tube, and spun down in a centrifuge for 8 minutes at 10,000 rpm. Next, two replicate 200 µl samples of the supernatant were used to quantify the amount of protein and carbohydrate using the coomassie dye protocol (CB-Protein assay; G-Biosciences) and a modified phenol-sulfuric acid protocol , respectively. Spectrophotometric readings were also obtained for experimental foods. Finally, regression plots were generated from spectrophotometric readings of experimental foods against the protein or carbohydrate content of these foods, and used to determine the protein and carbohydrate content of unconsumed food (linear fit: r^2^ = 0.95 and 0.97 for protein and carbohydrate, respectively). To determine whether ants were manipulating the macronutrient content of collected foods, the p:c ratio of unconsumed foods was compared to those of unmanipulated experimental foods.

**Results**

**Manipulation of nutrient content of unconsumed foods.**

**No-choice Experiments.** Most of both summer and fall colonies did not consume all collected food. The percentage of summer and fall colonies having unconsumed food was 93% and 83%, respectively. Season and treatment factors did not significantly affect whether colonies contained unconsumed food (nominal logistic likelihood ratio test: *X*^2^ < 1.50, df = 1, *P* = 0.221; *X*^2^ = 3.90, df = 4, *P* = 0.419, respectively). Whether colonies contained unconsumed food was marginally significantly affected by an interaction between season and treatment factors (*X*^2^ = 9.45, df = 4, *P* = 0.051). A number of summer colonies feeding on food p19:c57 did not contain any unconsumed foods. For those colonies containing unconsumed food, the amount of unconsumed food was significantly greater for summer colonies (ANOVA of log-transformed data: *F* _1, 52_ = 9.11, *P* = 0.004), and increased significantly as a function of the protein content of the food (*F* _4, 49_ = 3.29, *P* = 0.019). However, the quantity of unconsumed food was not significantly affected by an interaction between season and treatment factors (*F* _4, 49_ = 0.10, *P* = 0.983) (Fig. S1A). For all colonies, a significantly greater proportion of food collected by summer colonies remained unconsumed (F_1, 59_ = 6.58, *P* = 0.013), and as the protein content of the experimental food increased, a greater proportion of collected food remained unconsumed (F_4, 57_ = 4.29, *P* = 0.005). This latter trend was similar for both summer and fall colonies (F_4, 59_ = 1.29, *P* = 0.286) (Fig. S1B).

Spectrophotometric assays of unconsumed foods indicated that ants were manipulating the nutrient profile of experimental foods. The p:c ratio of unconsumed foods was significantly increased from that of most experimental foods (Table S2). The p:c ratio of unconsumed foods was significantly different between seasons (ANOVA of log-transformed data: *F*_1, 50_ = 4.16; *P* = 0.048). Additionally, the p:c ratio of unconsumed foods was significantly different across diets (*F*_4, 48_ = 4.50; *P* = 0.004), and a marginally significant season-by-diet interaction was observed (*F*_4, 48_ = 2.51; *P* = 0.056).

**Choice Experiment.** Most of both summer and fall colonies did not consume all collected foods. The percentage of summer and fall colonies having some unconsumed food was 94% and 89%, respectively. Whether colonies contained unconsumed food was not significantly affected by either season or treatment factors (nominal logistic likelihood ratio test: *X*^2^ < 0.01, df = 1, *P* = 0.999; *X*^2^ = 4.89, df = 2, *P* > 0.087, respectively), or by an interaction between these factors (*X*^2^ = 1.92, df = 2, *P* = 0.381). For those colonies containing unconsumed food, the amount of unconsumed food was significantly greater for summer colonies (ANOVA of log-transformed data: *F* _1, 27_ = 7.75, *P* = 0.010). However, the amount of unconsumed food was not significantly different across treatments (*F* _2, 27_ = 1.68, *P* = 0.205), or by an interaction between season and treatment factors (*F* _2, 27_ = 0.09, *P* = 0.911). For all colonies, the proportion of total collected foods that remained unconsumed was not significantly different between seasons (F_1, 30_ = 2.42, *P* = 0.130), nor was the proportion significantly different across treatments (F_2, 30_ = 2.78, *P* = 0.078). Additionally, there was no significant interaction between season and treatment factors (F_2, 30_ = 0.54, *P* = 0.588).

Spectrophotometric assays of unconsumed foods indicated that ants were manipulating the nutrient profile of experimental foods. The p:c ratio of unconsumed foods was significantly increased from that of the total combined collected foods (Table S3). The p:c ratio of unconsumed foods was similar for both summer and fall colonies (ANOVA of log-transformed data: *F*_1, 26_ = 0.80; *P* = 0.378). Additionally, the p:c ratio of unconsumed foods was similar across diet treatments (*F*_2, 26_ = 0.31; *P* = 0.734), and no significant season-by-diet treatment interaction was observed (*F*_2, 26_ = 0.12; *P* = 0.887).

1. Taylor KACC (1995) A Modification of the Phenol Sulfuric-Acid Assay for Total Carbohydrates Giving More Comparable Absorbances. Applied Biochemistry and Biotechnology 53: 207-214.
